# Reflectance spectroscopy as a promising tool for ‘sensing’ metals in hyperaccumulator plants

**DOI:** 10.1007/s00425-023-04167-3

**Published:** 2023-07-09

**Authors:** Imam Purwadi, Peter D. Erskine, Antony van der Ent

**Affiliations:** 1 Centre for Mined Land Rehabilitation, Sustainable Minerals Institute, The University of Queensland, Brisbane, QLD Australia; 2 Laboratory of Genetics, Wageningen University and Research, Wageningen, The Netherlands; 3 Laboratoire Sols et Environnement, INRAE, Université de Lorraine, Nancy, France

**Keywords:** Absorption band, Forest imaging, Herbarium specimens, Hyperaccumulator, Mineral exploration, Reflectance spectroscopy, Remote sensing

## Abstract

**Main conclusion:**

The VNIR reflectance spectra of nickel hyperaccumulator plant leaves have spectral variations due to high nickel concentrations and this property could potentially be used for discovery of these plants.

**Abstract:**

Hyperaccumulator plants accumulate high concentrations of certain metals, including manganese, cobalt, or nickel. Of these metals, the divalent ions of nickel have three absorption bands in the visible to near-infrared region which may cause variations in the spectral reflectance of nickel hyperaccumulator plant leaves, but this has not been investigated previously. In this shortproof-of-concept study, the spectral reflectance of eight different nickel hyperaccumulator plant species leaves were subjected to visible and near-infrared and shortwave infrared (VNIR-SWIR) reflectance spectrum measurements in dehydrated state, and for one species, it was also assessed in hydrated state. Nickel concentrations in the plant leaves were determined with other methods and then correlated to the spectral reflectance data. Spectral variations centred at 1000 ± 150 nm were observed and had R-values varying from 0.46 to 0.96 with nickel concentrations. The extremely high nickel concentrations in nickel hyperaccumulator leaves reshape their spectral reflectance features, and the electronic transition of nickel-ions directly contributes to absorption at ~ 1000 nm. Given that spectral variations are correlated with nickel concentrations it make VNIR-SWIR reflectance spectrometry a potential promising technique for discovery of hyperaccumulator plants, not only in the laboratory or herbarium, but also in the field using drone-based platforms. This is a preliminary study which we hope will instigate further detailed research on this topic to validate the findings and to explore possible applications.

**Supplementary Information:**

The online version contains supplementary material available at 10.1007/s00425-023-04167-3.

## Introduction

**Fig. 1:**
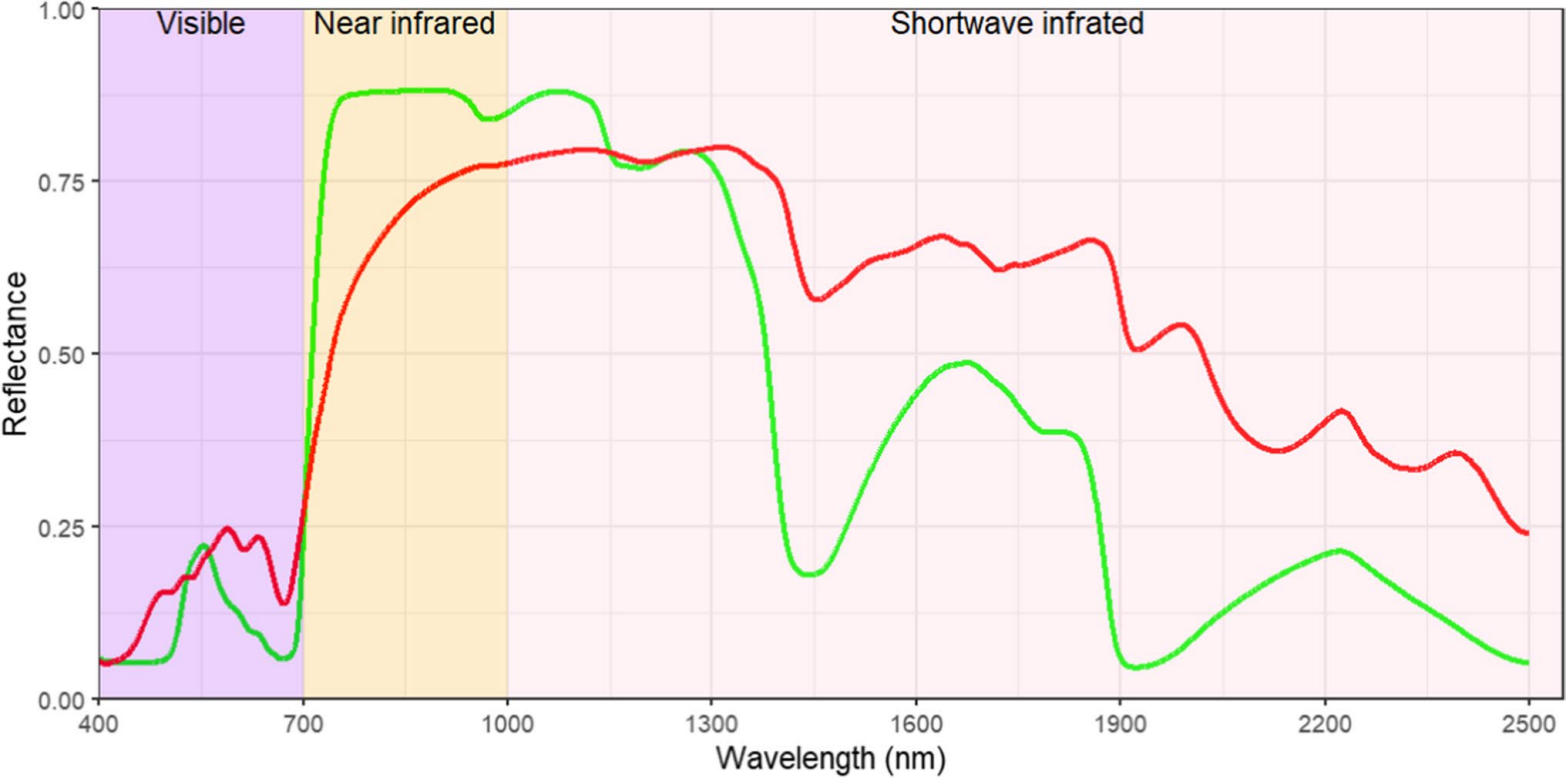
Spectral reflectance of *Melastoma malabathricum* leaf: green line  showing a spectrum of a sample in hydrated state and red line showing a spectrum of a sample in dehydrated state

A plant leaf has a distinct response on how it reflects or absorbs natural light from the sun. Chlorophyll absorbs almost all of the incoming visible light [400–700 nm (nm)] and reflects a small portion of the incident green light at around ~ 550 nm (e.g., Lodish et al. 2000; Johnson 2016), as shown in Fig. 1. However, chlorophyll does not absorb incident light in the near-infrared wavelength region (e.g., the 700–1000 nm range) (Knipling 1970). The internal structure of a leaf affects the reflectance of near-infrared light (Mestre 1935) with incident near-infrared light being scattered and some of it exiting through the top surface of the leaf, contributing to the total reflectance in this region (Knipling 1970; Gausman 1974; Ustin and Jacquemoud 2020). A *Citrus sinensis* leaf, for example, absorbs 90%, reflects ~ 10%, while it reflects 55%, transmits 40%, and absorbs 5% of the incident near-infrared light (Gausman 1985). In the shortwave near-infrared (SWIR) wavelength (1000–2500 nm) region, water content predominantly controls the reflectance of light (Allen et al. 1969). As the water content decreases, overall reflectance increases (Fig. 1). Four absorption bands at ~ 975 nm, 1200 nm, 1400 nm, and 1900 nm are caused by the vibrational bonds of water molecules: the combination of hydrogen–oxygen–hydrogen and the asymmetric hydroxyl stretch vibration (Danson et al. 1992). The high reflectivity of plant leaves in the near-infrared region is also caused by the lack of absorption in this part of the spectrum (Knipling 1970; Gausman 1974; Ustin and Jacquemoud 2020), whlist the cause of absorption bands in the visible and near-infrared (VNIR) reflectance spectrum is the electronic transition in molecules (van der Meer et al. 2012). Electronic transition involves bonding electrons, charge-transfer electrons, and d orbital electrons (Atkins et al. 2009). Plant dry biomass is mainly composed of 80-98% lignin, cellulose, and hemicellulose, and the the remainder are organic and inorganic minerals (Crawford 1981; Pasangulapati et al. 2012). Oxygen, carbon, and hydrogen are bonded together in different ratios to form lignin, cellulose, and hemicellulose, and absorption bands that result from the vibration of electrons in the bonding can be found in the far-infrared region (1000–20,000 nm) (Elvidge 1988; Curran 1989).

Plant leaves also contain other elements in large quantities (> 1000 μg g^−1^) such as calcium (Ca), potassium (K), sulphur (S), phosphorus (P), magnesium (Mg), and nitrogen (N) (Mengel et al. 2001; Dalcorso et al. 2014). Nitrogen is mostly found in the form of proteins, and the absorption bands due to the vibration of nitrogen–hydrogen bonding is present at wavelengths longer than 1000 nm (Elvidge 1988; Curran 1989). On the other hand, Ca, K, S, P, and Mg usually form ions or carboxyl groups (Mengel et al. 2001). VNIR radiation  can not ionize these elements, while the electronic transition in the carboxyl group bonding electrons is in the ultraviolet wavelength range (Evans and Price 1937). Plant leaves also contain transition metals so that d orbital electron transitions may occur. However, the concentration of the transition metals in normal plants is typically less than 100 μg g^−1^ (Mengel et al. 2001; Dalcorso et al. 2014), which is insufficient to generate absorption bands related to the d orbital electron transition.

A minority of plant species, known as hyperaccumulators, can attain extremely high concentrations of certain d-block transition metals into their shoots without suffering any toxicity symptoms (van der Ent et al. 2013). Hyperaccumulators includes those that contain at least 10,000 μg g^−1^ manganese; or 3000 μg g^−1^ zinc (Zn); or 1000 μg g^−1^ nickel (Ni), or 300 μg g^−1^ cobalt (Co), or copper (Cu) in their dry weight leaves (Baker and Brooks 1989; Reeves 2003; van der Ent et al. 2013). In a normal plant, a high concentration of these transition metals would rapidly lead to toxicity symptoms and plant death (Singh et al. 2016), and increase the reflectance of plant leaves experiencing metal-induced stress (Horler et al. 1980; Carter 1993). Hyperaccumulators spectral reflectance may be affected by the d orbital electron transition of metal complexes, but this has not yet been studied. Studying the reflectance spectroscopy of hyperaccumulator plants could help locate hyperaccumulator plant species as they are currently in danger of extinction due to habitat destruction due to land clearing and mining (Whiting et al. 2004; Erskine et al. 2012; Wulff et al. 2013). The pratical application of hyperaccumulator plant species varies from mineral exploration, microbiology, phytoremediation, to phytomining (Brooks and Brooks 1998; Reeves 2003). A total of 721 identified plant species have been documented to date as hyperaccumulators (Reeves et al. 2017), but only two species are being used for commercial (Ni) phytomining currently (Chaney et al. 2018). The lack of mass screening tools has impeded the discovery of hyperaccumulator plants (van der Ent et al. 2019), but in recent years, handheld X-ray fluorescence (XRF) spectrometer instrumentation has been used to perform systematic screening of herbarium specimens to identify new hyperaccumulator plant species (Gei et al. 2018; van der Ent et al. 2019). However, the XRF method for screening hyperaccumulator plants using a portable XRF device is limited to one measurement at one time and generally require the users to have a radiation license . Remote sensing techniques may be suitable for locating hyperaccumulator plant species, even in dense and inaccessible vegetation at the individual species to landscape-scale level (Gupta 1991). However, methodological studies are required to better understand the reflectance spectroscopy of hyperaccumulator plant leaves before a suitable remote sensing technique could be developed

Therefore, this exploratory study aims to test and interpret spectral variations observed in the reflectance spectroscopy of hyperaccumulator plant leaves under controlled conditions. As such, we aim to verify whether this technique has potential for differentiating between normal plants and hyperaccumulator plants and to assess the limitations of sensing hyperaccumulator plants using reflectance spectrometry.

## Materials and methods

### Plant selection and reflectance measurements

**Fig. 2:**
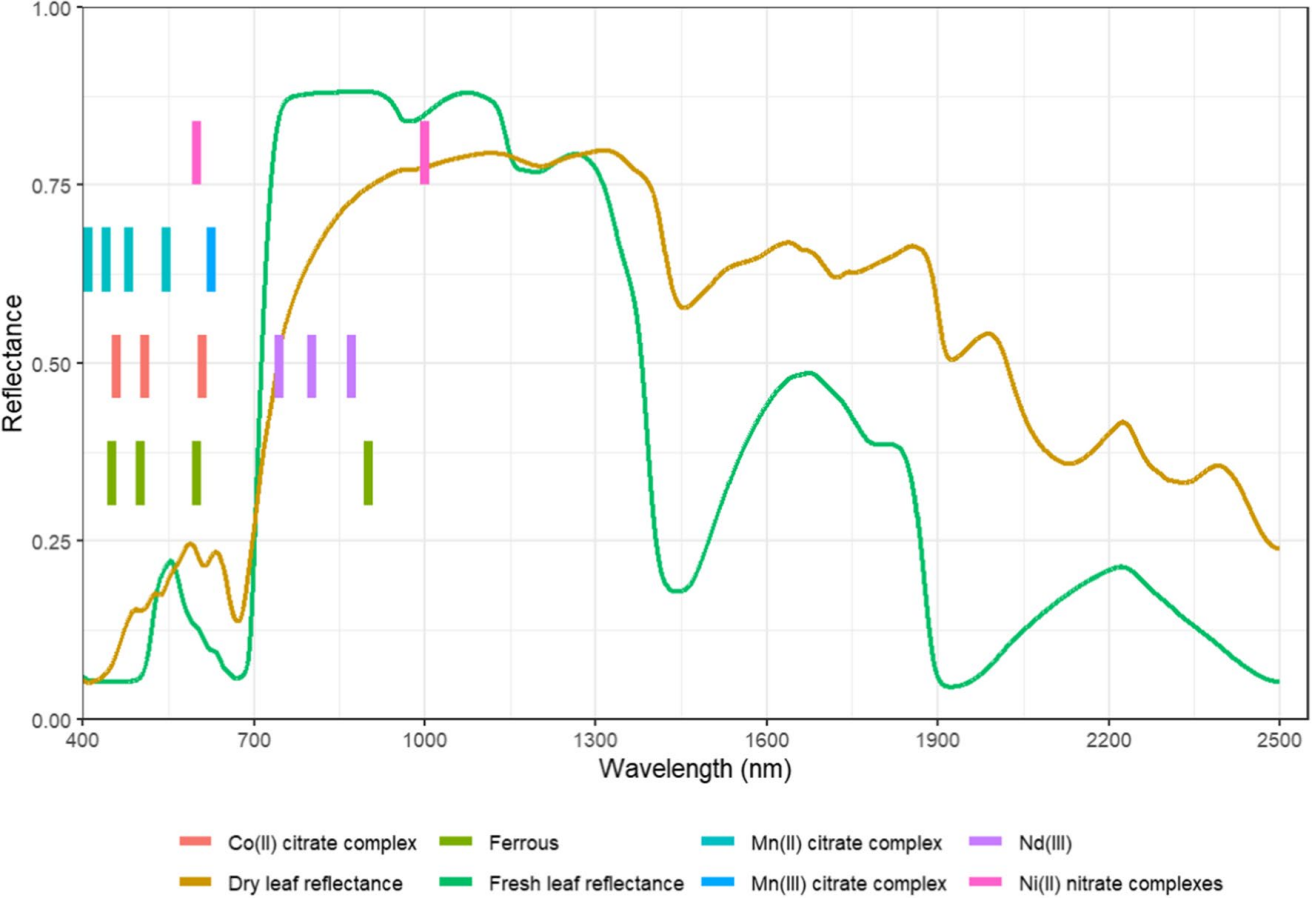
Several possible positions (indicated by vertical lines derived from Suppl. Table S1) of absorption bands due metal complexes in the VNIR reflectance spectra of plant leaves

The first criterion for selecting leaf samples was to focus on elements that may have VNIR spectral features due to the presence of high concentrations of a specific hyperaccumulated metal. Transition metals and rare earth elements (REEs) are known to be spectrally active in absorbing incident VNIR light (Cotton 2006; Sridharan 2016). We did not have access to REE hyperaccumulator samples and therefore, we focussed on Ni hyperaccumulator plant leaves. As summarised in Suppl. Table S1 and shown in Fig. 2, Ni^2+^ was suspected to absorb incident VNIR light and has absorption bands that do not overlap with the absorption bands of water and chlorophyll. Moreover, Ni concentrations in hyperaccumulators can reach several wt% levels, such that sufficient effects were detected in the spectrum. We measured *Berkheya coddii* in hydrated states and leaves of various other Ni hyperaccumulators (*Glochidion bambangan, Glochidion panataran, Phyllanthus rufuschaneyi, Rinorea bengalensis, Rinorea javanica, Actephila alanbakeri* and *Walsura pinnata)* originating from our archive of sample materials  in dehydrated state. The VNIR spectra were collected using an ASD FieldSpec 3 instrument with contact probe and internal high-intensity lamp in a dark room. The ASD instrument has a spectral range from 350 to 2500 nm covered by three spectrometers (VNIR: 350–1050 nm, SWIR 1:1050–1800 nm, and SWIR 2: 1800–2500 nm). Calibration was done every ten samples by measuring a 100% white reference. After measuring the hydrated leaves of *B. coddii* the leaves were dried ar 60 ºC for 48 h and measured again in dehydrated state.

### Elemental concentration quantifications

Inductively coupled plasma atomic emission spectroscopy (ICP-AES) was used to determine the Ni concentrations of *B. coddii* leaves, after weighing and acid digestion(pre-digestion in 2 mL of 70% HNO_3_for 48 h and 2 hrs at 120 ºC in Thermo Scientific™ block heater). The digestates were brough to volume (10 mL) and analysed with a Thermo Scientific iCAP 7400 instrument for Ni with yttrium internal addition to compensate for interferences due to matrix variations. Quality controls were performed by measuring certified reference material (Sigma-Aldrich Periodic Table mix 1 TraceCERT^®^, 33 elements, 10 mg L^−1^ in HNO_3_), matrix blanks, and internal standards.

Dehydrated leaves of *G. bambangan, G. panataran, P. rufuschaneyi, R. bengalensis, R. javanica, A. alanbakeri,* and *W. pinnata* were measured using XRF, which has the main benefit that identical samples could be meastred directly after VNIR measurement. The XRF analysis used a Thermo Fisher Scientific Niton XL3t 950 GOLDD+ analyser with the in-built ‘Soils Mode’ and ‘Main filter’ configurations for 30 s. The XRF spectra were extracted and processed in GeoPIXE software using a pipeline developed for plant leaves (Purwadi et al. 2022).

### Statistical analysis

**Fig. 3:**
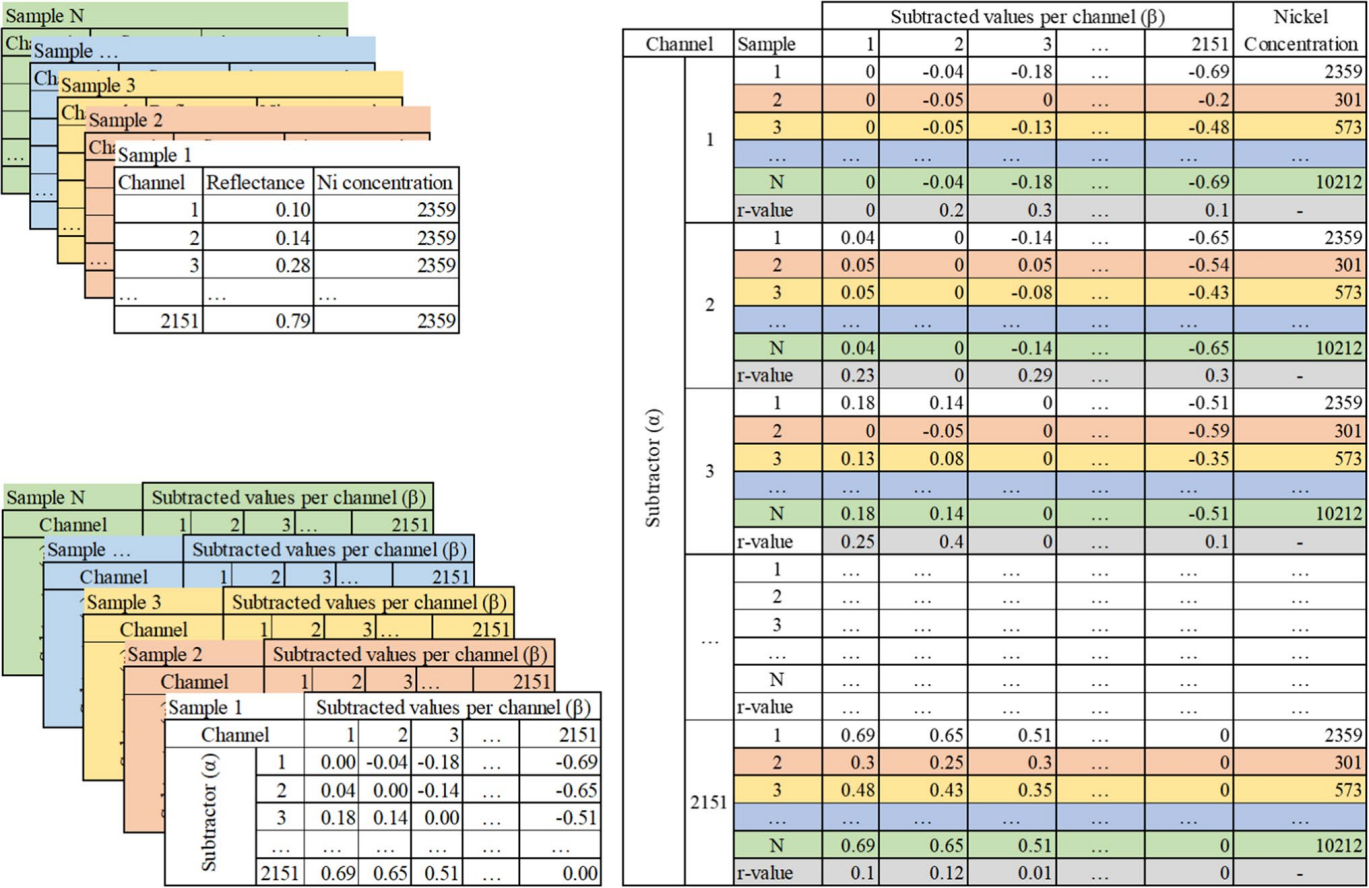
Each sample consists of two components: reflectance values (2151 channels) and Ni concentrations (Top left). Variations in reflectance values are calculated by subtracting all channel values to one channel generating a 2151 × 2151 matrix (Bottom left). The 2151 × 2151 matrix of all samples are stacked together and sorted per subtractors and related to Ni concentrations. The outputs, r values, are plotted as a map, with subtractor channels (*α*) in x axis, subtracted channels (*β*) in y axis, and *R* values at a coordinates of (*α*, *β*)

In this study, 1 sample has 2 components: the Ni concentration and the reflectance values consisting of 2151 contiguous bands or channels between 350 and 2500 nm (Fig. 3 top left). The aim of statistical analysis was to assess the relation between spectral variations and Ni concentrations of Ni hyperaccumulator plant leaves. Differences in reflectance values across the channels result from the absence or presence of specific absorption features of a molecule (Huete 2004), and to relate the differences to Ni concentration, the reflectance values of all 2151 channels were subtracted each channel one by one, thus producing a 2151 × 2151 matrix for one sample (Fig. 3 bottom left). The 2151 × 2151 matrix for each sample was stacked together to form a 3D matrix with a dimension of N × 2151 × 2151 (where N is the total number of samples). The 3D matrix was grouped based on the subtractor channel (*α*), and the subtracted values per channel (*β*) was correlated to Ni concentrations (Fig. 3 right). The final output are correlation maps where the x-axis is α, and the y-axis is β, and the values at the coordinate (*α*, *β*) were *R* values with a 95% confident level. This process was performed using R version 4.1.1 (R Core team 2021).

### Comparison to the spectral reflectance of ‘normal’ dehydrated plant leaves

Comparing the VNIR spectral reflectance of leaves of normal and hyperaccumulation plants can provide valuable information on useful wavelengths to distinguish both groups. In this study, we compared the spectral reflectance of dehydrated hyperaccumulator leaves (286 spectra) acquired during this study to ‘normal’ dry leaves (553 spectra) reported by Meerdink et al. (2019). with both studies using the same instrument. The dehydrated leaves were chosen because results produced from this study could be used immediately, for example, to identify hyperaccumulator plants from measuring herbarium specimens. All of the spectra were subtracted by its reflectance value at 1350 nm. as at this wavelength (1350 nm), a peak is observed, as shown in Fig. 1. After that, all bands or channels were fed into a simple random forest classification. Prior to the classification, the datasets were divided into 75% for training and 25% for validation. Finally, a feature importance that gives a score to each wavelength was calculated. The highest score of the feature importance indicates the most useful wavelength in classifying 'normal' versus hyperaccumulator plant leaves.

## Results

### Nickel concentrations and spectral reflectance properties

**Fig. 4:**
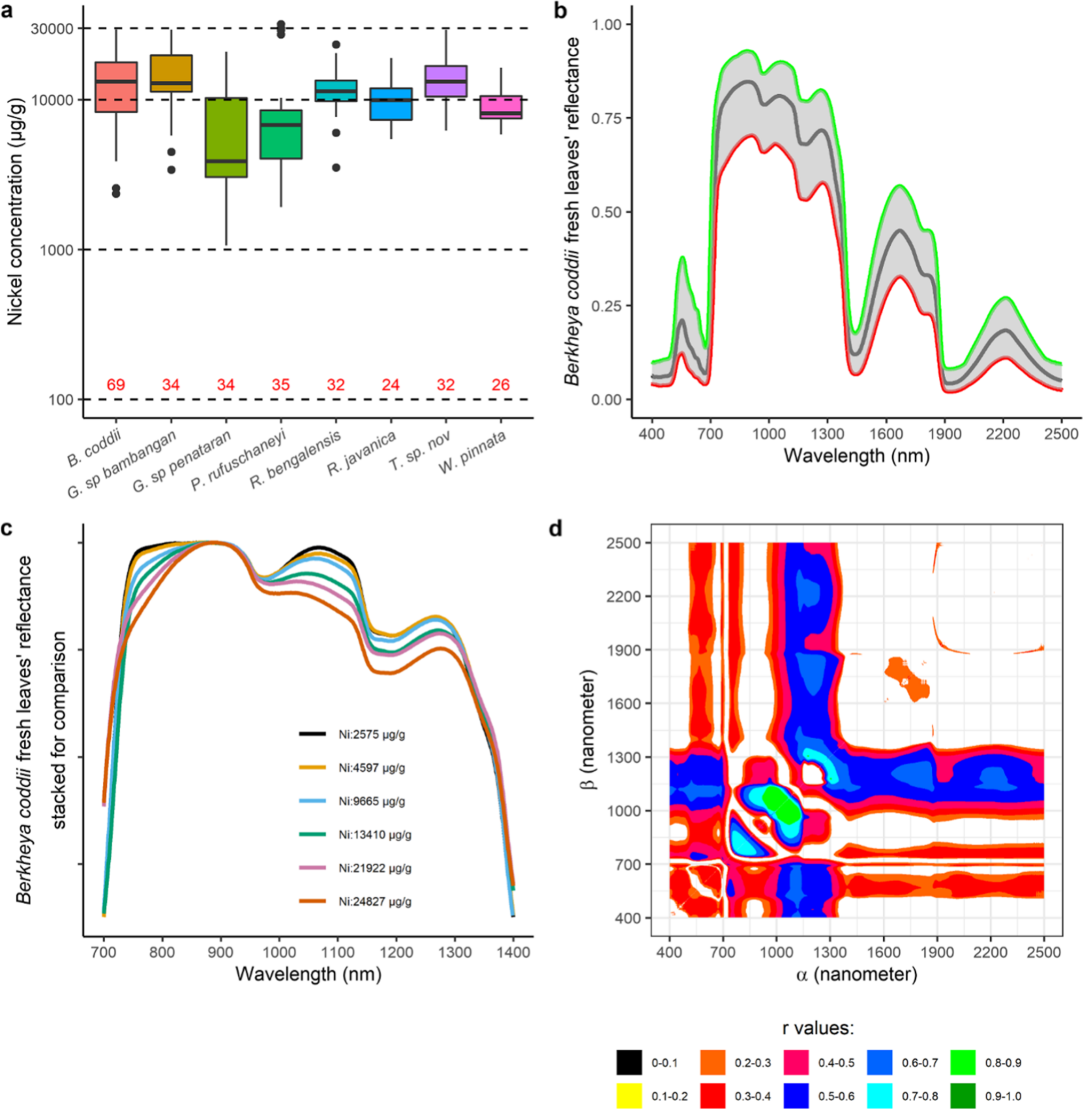
Nickel concentrations in the leaves of 8 Ni hyperaccumulator plant species (**a**). All samples have >1000 μg g^−1^ Ni. Minimum (red), mean (black), and maximum (green) of *Berkheya coddii* hydrated leaf reflectance spectral values in (**b**) show variations with changes in response to Ni concentrations (**c**). A map (**d**) provides information on the correlations between Ni concentrations and differences in one band (*α*) to the remaining spectral bands (*β*). Correlation values with confident levels less than 95% are masked

**Fig. 5:**
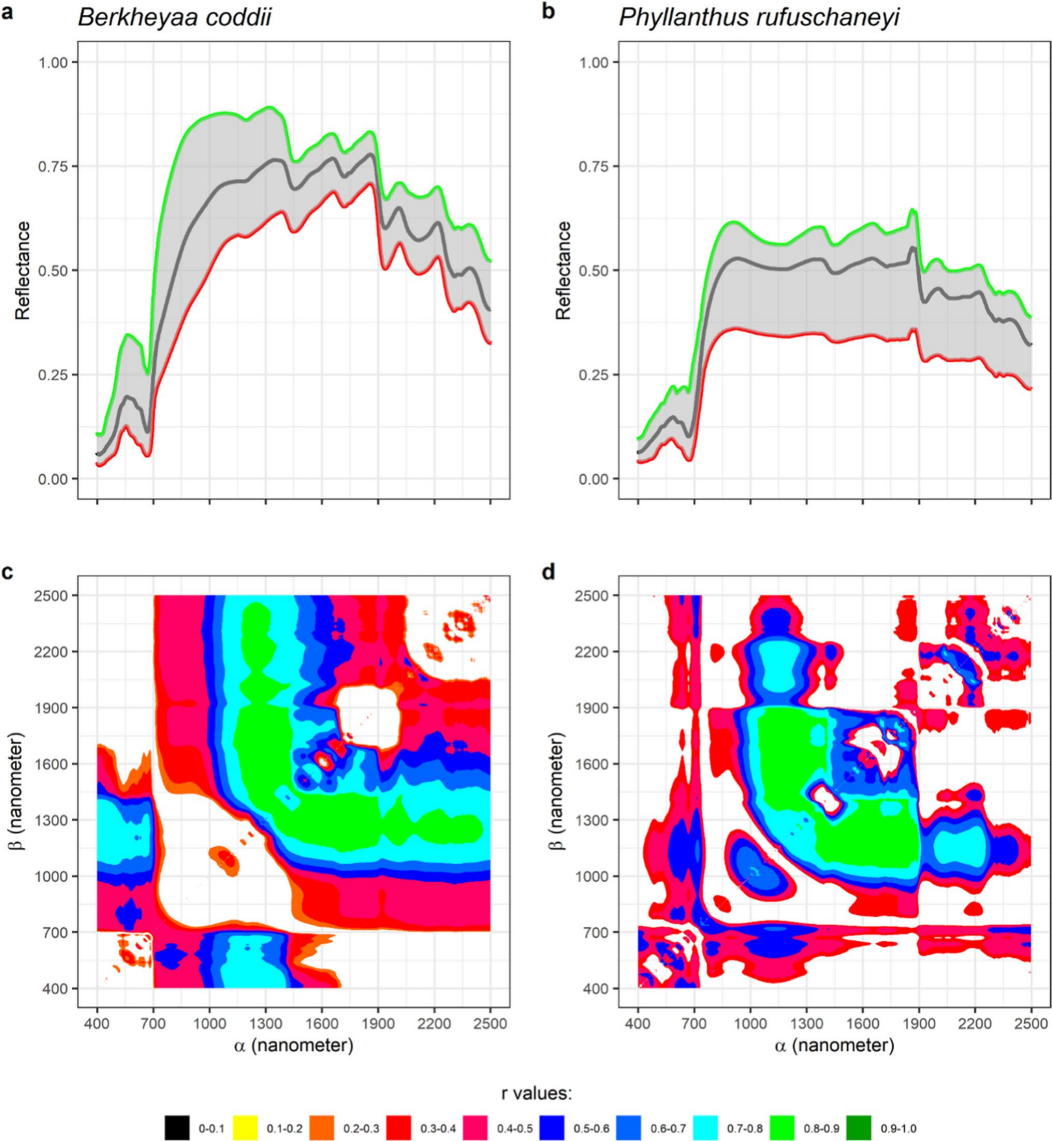
**a**, **b** Minimum (red), mean (black), and maximum (green) of *Berkheya coddii* and *Phyllanthus rufuschaneyi* dehydrated leaf reflectance spectra. Correlation maps between spectral variations and Ni concentrations of *Berkheya coddii* (**c**) and *Phyllanthus rufuschaneyi* (**d**)

**Fig. 6:**
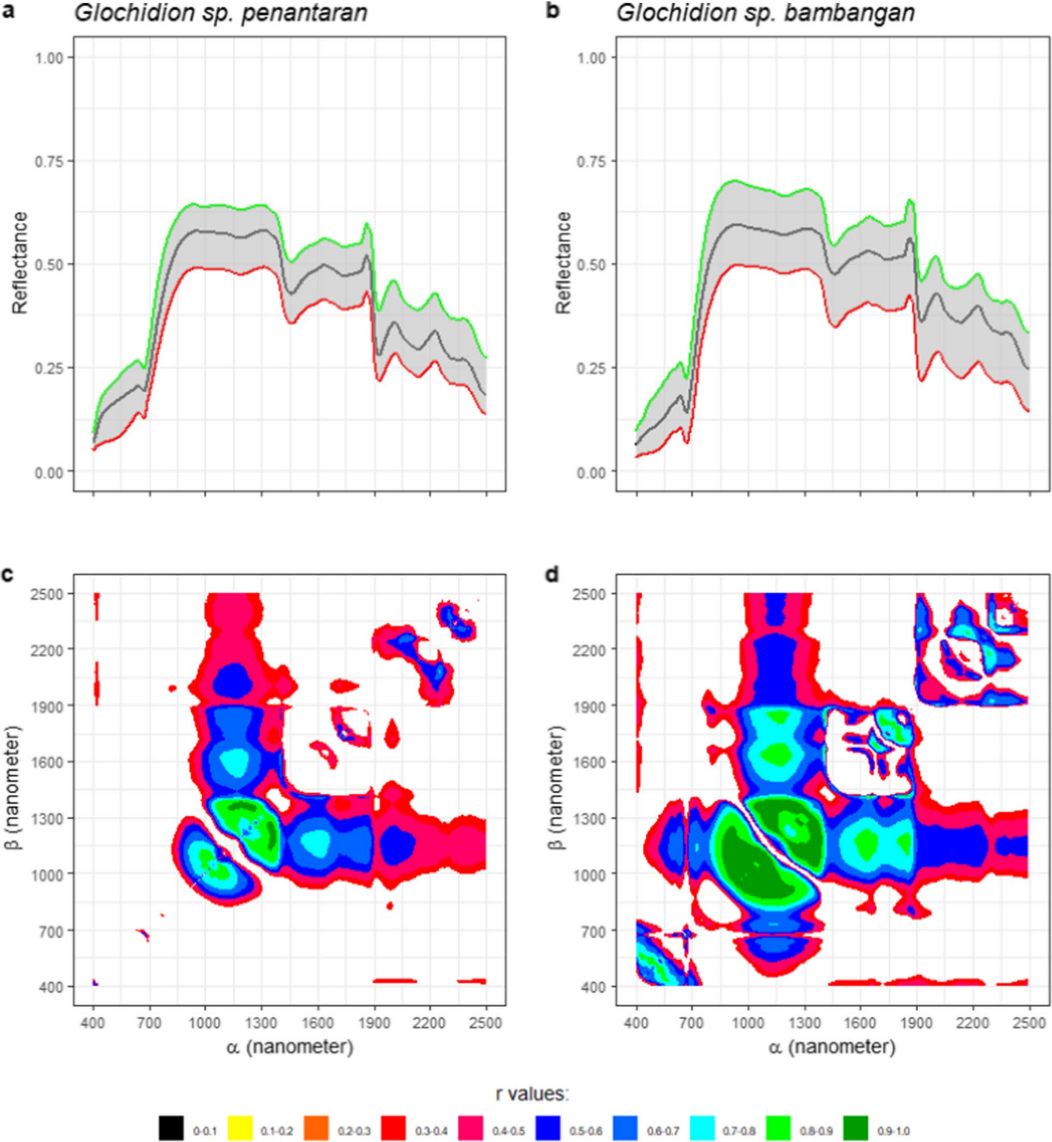
**a**, **b** Minimum (red), mean (black), and maximum (green) of *Glochidion ‘panataran’* and *Glochidion ‘bambangan’* dehydrated leaf reflectance spectra. Correlation maps between spectral variations and Ni concentrations of *Glochidion ‘panataran’* (**c**) and *Glochidion ‘bambangan’* (**d**)

**Fig. 7:**
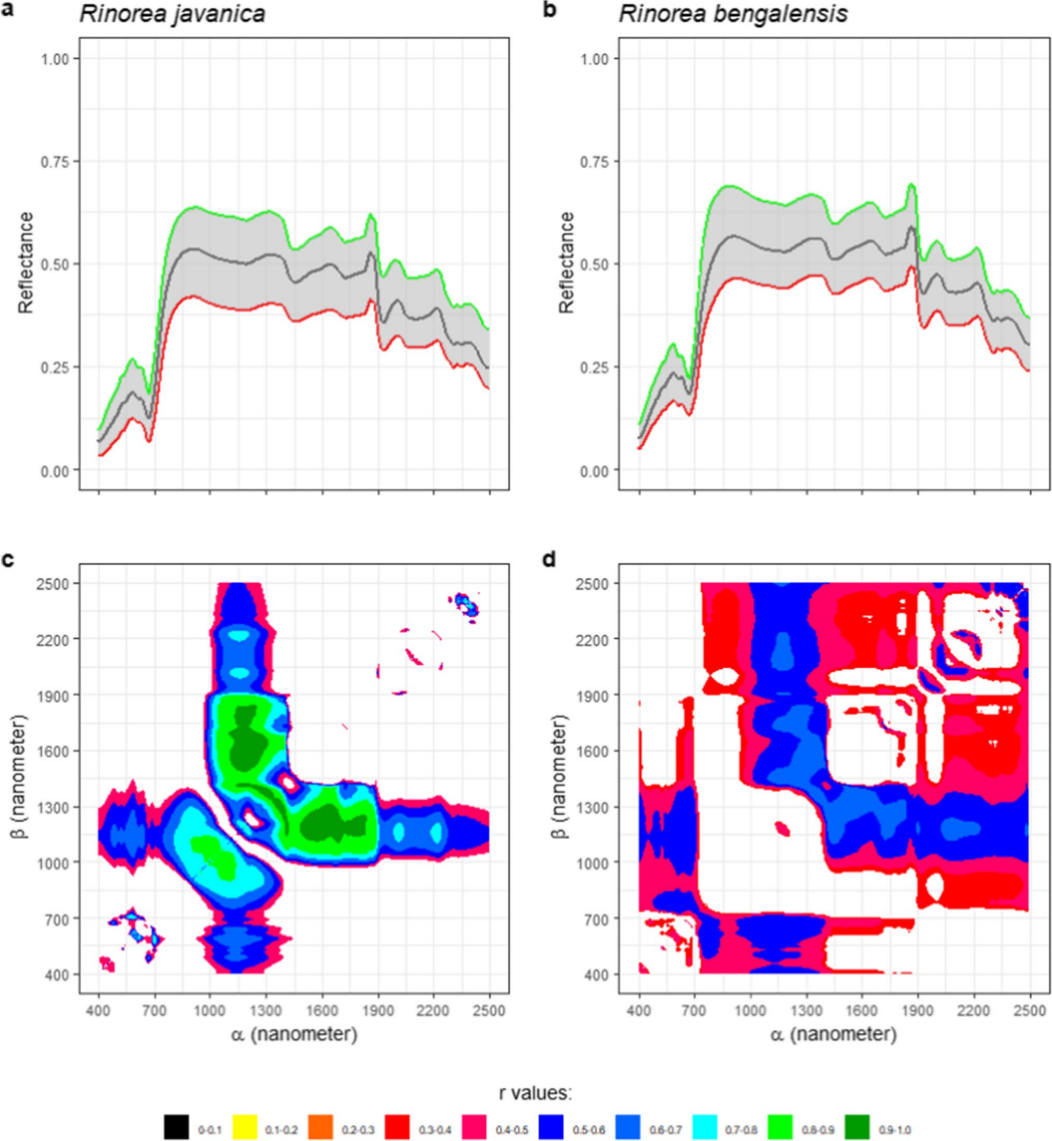
**a**, **b** Minimum (red), mean (black), and maximum (green) of *Rinorea javanica* and *Rinorea bengalensis* dehydrated leaf reflectance spectra. Correlation maps between spectral variations and Ni concentrations of *Rinorea javanica* (**c**) and *Rinorea bengalensis* (**d**)

Nickel concentrations of 8 hyperaccumulator plant species are shown in Fig. 4a. The number of samples per species ranged from 24 samples (*R. javanica*) to 69 samples (*B. coddii*). No samples contained Ni concentrations below 1000 µg g^−1^ with the average concentration being approx. 10,000 µg g^−1^. From Fig. 4b, variations in the spectral reflectance of *B. coddii* hydrated leaves with varying Ni concentrations an be observed as highlighted in Fig. 4c. One local maxima was usually found around 1100 nm, and the reflectance values around this region drops as Ni concentrations increase. In the case of the spectral reflectance of dehydrated leaves, the water absorptions at ~ 900 nm and 1200 disappeared, and a broad absorption band appeared at ~ 1100 nm (Figs. 5, 6, 7).

### Statistical correlations between spectral variations and Ni concentrations

**Fig. 8:**
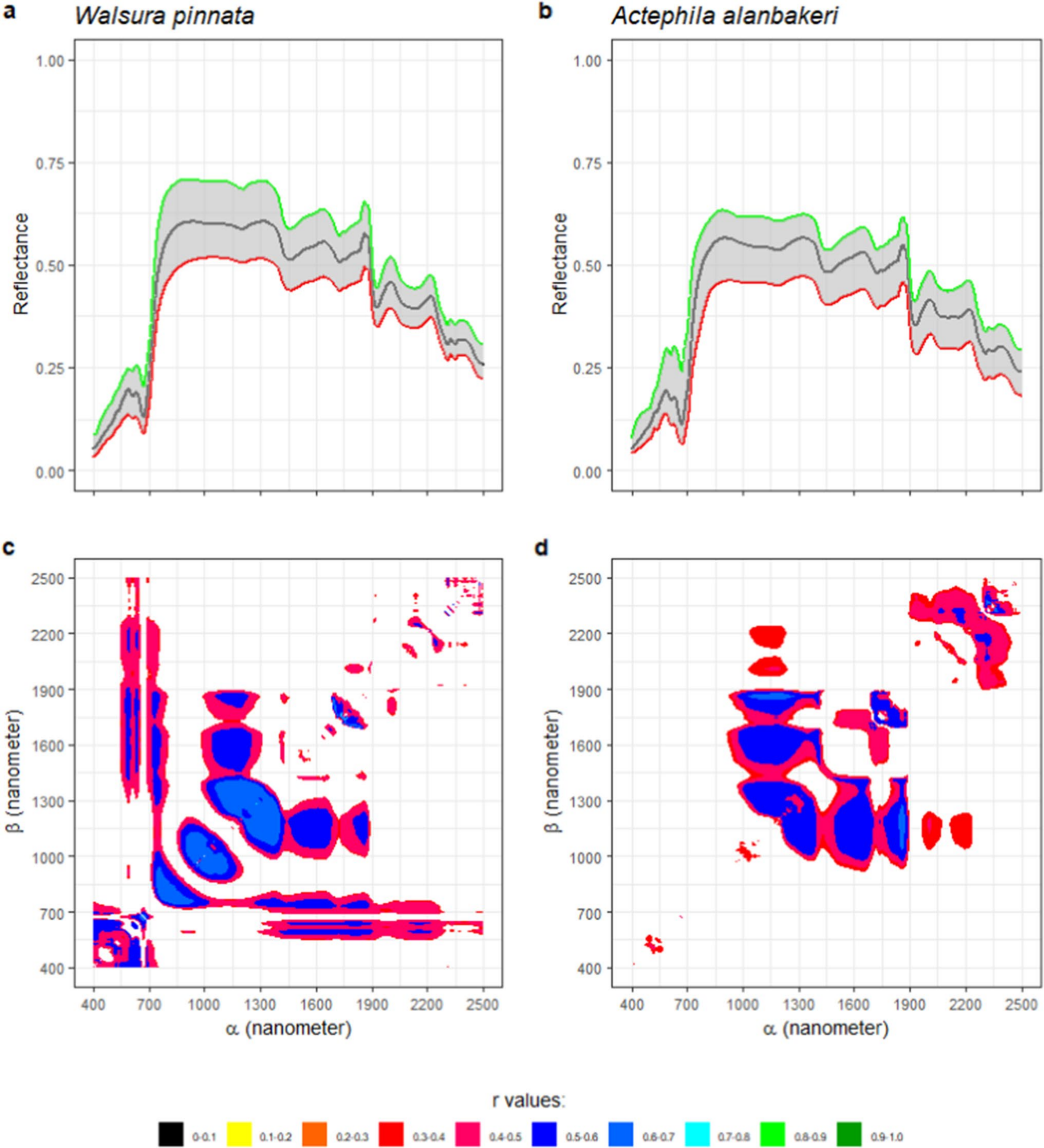
**a**, **b** Minimum (red), mean (black), and maximum (green) of *Walsura pinnata* and *Actephila alanbakeri* dehydrated leaf reflectance spectra. Correlation maps between spectral variations and Ni concentrations of *Walsura pinnata* (**c**) and *Actephila alanbakeri* (**d**)

Spectral variations were defined as differences in the reflectance value of one band to the remaining 2150 bands. The differences were calculated for all 2151 bands, thus producing a 2151 × 2151 matrix per sample. The matrixes then were related to samples’ Ni concentrations, and the results are shown in Figs. 4, 5, 6, 7 and 8. The one dataset for hydrated leaves, *B. coddii*, had correlation values as high as R 0.9 in spectral variations centred around 1000 nm. An elongated pattern indicating correlation values ranging from R 0.4 to 0.8 was observed when the subtractors were between 1000 and 1300 nm, and the subtracted bands were > 1300 nm. The correlation maps between the spectral reflectance of *B. coddii* dehydrated leaves and Ni concentrations revealed different patterns. Whilst no strong correlations were observed centred around 1000 nm, the elongated pattern of relationships in the region of 1000–1300 nm and the region > 1300 nm was overall higher (Fig. 5a, c). *Phyllanthus rufuschaneyi, G. ‘panataran’, G. ‘bambangan*,’ and *R. javanica* had similar spectral correlation patterns, but different correlation coefficient values. Unlike *B. coddii*, the elongated pattern only showed a strong correlation up to 1900 nm. This correlation region at ~ 1000 nm was observed in hydrated, but not in dehydrated leaves of *B. coddii,* and was found in all four tested species with even stronger correlation coefficients (up to R 0.9) in *G. ‘bambangan’.* In the case of *R. bengalensis, W. pinata,* and *A. alanbakeri*, weaker correlations were found, up to R 0.7, but the patterns in the correlation maps was similar.

### Classification of spectral classes of hyperaccumulator vs. normal plants

**Fig. 9:**
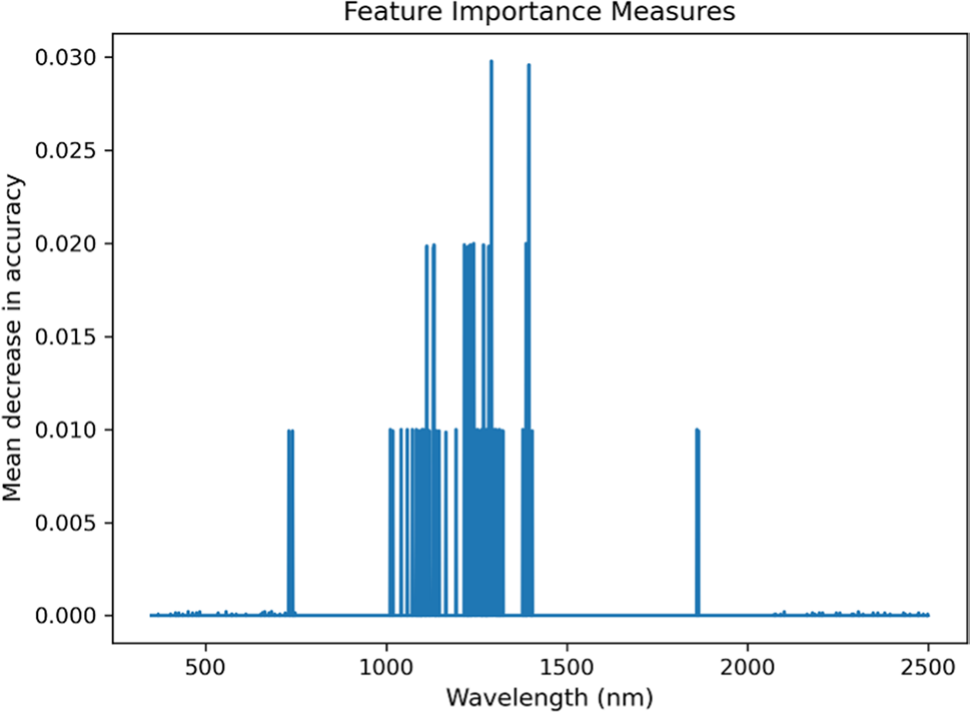
The feature importance of each band used for differentiating ‘normal’ plants from hyperaccumulator plants. High numbers indicate a high contribution of the specific band in improving the accuracy

A simple random forest model was trained to distinguish hyperaccumulator plants from normal plants using their reflectance recorded in 2151 bands. The model did not misclassify any validation sample, thus producing 100% accuracy. Figure 9 shows the feature importance for each band, and bands at the wavelength region between 1000 and 1500 nm are the major contributors in achieving this accuracy. This region coincides with high correlation area between spectral variations and Ni concentrations shown in Figs. 4, 5, 6, 7 and 8.

## Discussion

The divalent ions of Ni spectrally absorb light in the wavelength region between 300 and 1200 nm centred at ~ 360 nm, ~ 600 nm, and ~ 1000 nm that shifts depending on the ligands (Goldcamp et al. 2003). Relationships between Ni concentrations and absorptions at shorter wavelengths (< 700 nm) were in general low, most likely due to masking by chlorophyll absorption bands. Meanwhile, the results reveal relationships between Ni concentrations and absorption bands at 1000 ± 150 nm that were consistently present, albeit varying in degrees: *A. alanbakeri* (up to R 0.46), *R. bengalensis* (up to R 0.5), *P rufuschaneyi* (up to R 0.75), *W. pinnata* (up to R 0.79), *G. ‘panataran’* (up to R 0.83), *R. javanica* (0.86), *G. ‘bambangan’* (up to R 0.96). The maps show that not only the pattern, but also the correlation coefficient have variations, and those variations may be attributed to many different factors. The main factor determining the position of these absorption bands are the ligands of Ni^2+^ (Goldcamp et al. 2003) and Ni^2+^ in hyperaccumulator plant species is typically complexed with citrate (van der Ent et al. 2017). Carboxylic acids have absorption bands between 1600 and 1800 nm and between 2200 and 2500 nm that originate from stretching modes of carbon–hydrogen and oxygen–hydrogen (Türker-Kaya and Huck 2017; Grabska et al. 2017; Beć et al. 2020). Therefore, the absorption bands in the different hyperaccumulator species tested were expected to be similar. However, numerous factors may affect the position and shape of the absorption bands. For example, the weak bonding between Ni^2+^ and citrate complex can cause an impure ligand environment with nitrogen or sulphur donors (van der Ent et al. 2017). In the wavelength region of 700–1300 nm, internal leaf structures control how incident light is reflected, transmitted, and absorbed (Knipling 1970). Therefore, it is not surprising when an intact leaf of different Ni hyperaccumulator species responds differently when interacting with incident light. In the case of *B. coddii*, the leaves had indications of being photo-damaged when exposed to the light source of instrument within 2 s. Elongated patterns along the x and y axes are also observed with wavelength regions between 1000 and 1300 nm act as a symmetric line of the patterns. The divalent ions of Ni have three absorption bands arising from electronic transitions, and the three absorptions are found in the VNIR wavelength range. As the wavelength gets longer, it contains less energy, thus, not enough to eject an electron out of an atom or molecule to initialise electronic transitions. However, instead of electronic transitions, vibrational processes are dominant in longer wavelength in which the energy of incident light is absorbed by a molecule, causing bonds within the molecule to stretch and bend (Clark 1999). The elongated patterns observed in all correlation maps, also share a common behaviour in which they can be divided into two sections: the short wavelength region between 1300 and 1900 nm and the long wavelength region between 1900 and 2500 nm. The shorter wavelength region has a stronger relationship with Ni concentrations than the longer wavelength region. One possible explanation is that spectral variations in the long wavelength region are mainly affected by strong water absorption bands, and significant overlapping absorption bands occur in this region (Grabska et al. 2017). A random forest classification is a simple classification method built upon an ensemble of decision trees. In this study, 100 decision trees were trained to distinguish 'normal' plant leaves from hyperaccumulator plant leaves based on the spectral reflectance of the dehydrated leaves. During the training, each decision tree is asked a true or false question whether the spectrum belongs to a 'normal' or hyperaccumulator plant. During the validation processes, 100 decision trees are tested against validation datasets to answer the same question, and no spectrum was misclassified, thus producing 100% accuracy. As shown in Fig. 9, it is clear that bands between 1000 and 1500 nm are the major contributors. This aligns with Figs. 4, 5, 6, 7 and 8, that revealed that spectral variations occur in this region with high correlations with Ni concentrations. This further supports the observation that these spectral variations of Ni hyperaccumulator plant leaves are distinct and unique.

### Conclusion and future perspectives

Nickel^2+^ ions absorb radiation in the VNIR-SWIR region, and coupled with the high prevailing concentrations of Ni in hyperaccumulator plant leaves, spectral variations in hyperaccumulator plant leaves are plausible. This study shows that the spectral variations are correlated with Ni concentrations, and that spectral variations around 1000 nm are linked to Ni^2+^. The VNIR-SWIR reflectance spectrometer can measure these spectral features, which makes it a promising technique for discovery hyperaccumulator plants. Potential applications are not limited to laboratories and herbaria but could be extended to the field using a hyperspectral camera mounted on drone or aircraft platforms. The current state of the art of drone hyperspectral sensors in their spatial and spectra resolution enables to acquire reflectance spectra of a single leaf, given that these sensors can have spectral resolutions of 1.85 nm and spatial resolution < 0.1 m (Jaud et al. 2018) This compares to aircraft hyperspectral data that typically have 10–20 nm spectral resolution between and 2 and 10 m spatial resolution (Cocks et al. 1998).

This short study is clearly preliminary in nature and its findings need substantial validation and investigation to elucidate the nature of the spectral features found in relation to Ni in (hyperaccumulator) leaves. If fully validated, VNIR reflectance spectroscopy could offer many benefits over other methods used to discover hyperaccumulator plants. This includes a reduction in the cost and time of collecting samples from the field by measuring existing herbarium specimens with a VNIR spectrometer for discovery of novel hyperaccumulators. An additional benefit is that reflectance spectroscopy does not require radiation safety permits as is required for the use of XRF instrumentation. Furthermore, aircraft hyperspectral data has been collected in many areas around the world, including from metalliferous soils (Ong et al. 2014; Jet Propulsion Laboratory 2019) that are waiting to be data-mined, and there is a higher chance to find hyperaccumulators on metalliferous soils (Reeves et al. 2018). Validation of VNIR reflectance spectroscopy for discovering hyperaccumulator plants includes procedures to metal absorption bands overlapping with chlorophyll absorption bands (Fig. 2). Extracting chlorophyll from leaf tissue usually involves washing leaves in ethanol, methanol, pyridine, and acetone (Strain and Svec 1966), and potentially dissolves the metals contained within the leaf (e.g. Artwell et al. 2017), therefore, diminishing the likelihood of detecting relevant absorption bands. Furthermore, an algorithm to automatically classify hyperaccumulator and non-hyperaccumulator plants based on their reflectance has not been developed yet. Finally, it is unknown whether the VNIR spectroscopy method can be used for quantitative or qualitative analysis. Several metals have overlapping absorption bands and the XRF spectroscopy technique may be used to identify the metal causing spectral features in the VNIR data. Differences in ligands shift the position of absorption bands, and Raman spectroscopy could also be used to characterize this chemical variation (Heredia-Guerrero et al. 2014).

#### *Author contribution statement*

IP, PDE and AVDE: conceived of the presented idea. IP developed the theory and performed the analysis. PDE and AVDE: verified and aided in interpreting the results. IP, PDE, and AVDE: discussed and wrote the manuscript.

## Supplementary Information

Below is the link to the electronic supplementary material.

Supplementary file1 (PDF 229 KB)

## Data Availability

The datasets generated during and/or analysed during the current study are available from the corresponding author on reasonable request.

## Abbreviations

SWIR: Shortwave infrared
VNIR: Visible near infrared
XRF: X-ray fluorescence
