# Supplementary material for: Reflectance spectroscopy as a promising tool for ‘sensing’ metals in hyperaccumulator plants
Source: Planta. 2023 Jul 9;258(2):41. doi: 10.1007/s00425-023-04167-3 (PMC10329965; doi:10.1007/s00425-023-04167-3)
Supplement: Supplementary file 1 — Supplementary file1 (PDF 229 KB) [file 425_2023_4167_MOESM1_ESM.pdf]

Supplementary material

Reflectance spectroscopy as a promising tool for 'sensing' metals in hyperaccumulator plants

Imam Purwadi[1], Peter D. Erskine[1], Antony van der Ent[1,2,3*]

[1]Centre for Mined Land Rehabilitation, Sustainable Minerals Institute,
The University of Queensland, Queensland, Australia.

[2]Laboratory of Genetics, Wageningen University and Research, The Netherlands.

[3]Laboratoire Sols et Environnement, INRAE, Université de Lorraine, France.

*Corresponding author: A. van der Ent (antony.vanderent@wur.nl)

Table S1 The form of the first-row transition metals in plants.

	**Forms in plants**
Ti	Ti may form Ti^{4+}-organic acid complex such as ascorbic acid and citric acid to translocate Ti easily (Broadley et al., 2007), and thus has a d^0 configuration, so no transition is possible.
V	Vanadate (V^{5+}+O_4^{8-}) is analogue to phosphate (PO_4^{3-}) and play a role as counterions for Deoxyribonucleic acid and Ribonucleic acid (Akabayov et al., 2014; Crans et al., 2004), and inhibits plant enzyme (Kasai et al.1999). Similar to Ti^{4+}, V^{5+} has a d^0 configuration, so absorption bands are not expected.
Cr	Cr is very toxic for plant (Huffman & Allaway, 1973), and plants take Cr in the form of Cr^{4+}, and once entering the root, Cr^{4+} seems to be reduced to Cr^{3+}, thus accumulated in roots due to its immobility (Shanker et al., 2005). Therefore, no absorption bands related to Cr is expected to appear in the spectral reflectance of a leaf due to low concentration.
Mn	Mn^{2+} is the most stable valency (Lucchini et al., 2015), and in plant tissues, Mn presents as Mn^{2+} soluble (Blamey et al., 2018). In seven-Mn (hyper)accumulator leaves, Mn^{2+} forms a complex with either malate acid or citrate acid (Fernando et al., 2010). Mn(II) citrate complex is a d^5 configuration that is Laporte and Spin forbidden, so its absorption bands are weak, while Mn(III) citrate complex is a d^4 configuration and one absorption is expected due to $^5T_{2g} \rightarrow {}^5E_g$ transition that is centred at ~625 nm (Jones et al. 2019). The band of the Mn(III) citrate complex is coincided with the absorption band of chlorophyll, making it tricky to sense them unless chlorophyll is extracted from the leaves.
Fe	Fe is found in the form of Fe divalent and trivalent ions (Oven et al., 2002), and predicted to have citrate as its ligand (Lopez-Millan et al., 2000). Fe^{3+} has the same d^5 configuration as Mn^{2+}.
Co	Co^{2+} are absorbed from the soil (Palit et al., 1994) and appeared to form a complex with organic acid (Collins et al., 2010). In the Co hyperaccumulator *Crotalaria cobalticola*, Co is bound to citric acid and cysteine acid (Oven et al., 2002), and Co (II) citrate complex has been reported to have three absorption bands peaked at 458 nm, 508 nm, and 610 nm (Matzapetakis et al. 2000). Similar to Mn^{3+}, the absorption bands of the chlorophyll most probably mask these bands.
Ni	Ni^{2+} is a constituent of an enzyme (Mishra and Kar, 1974). In a Ni hyperaccumulator plant, *Alyssum murale*, Ni is bound primarily to malate, while in six accumulator plant, Ni is found in the form of citratonickelate complex (Lee et al., 1977). Ni^{2+} has a d^8 configuration, so three absorption bands are expected. Six different Ni^{2+} nitrate complexes have reported to have three absorption bands peaked at ~360 nm, ~600 nm, and ~1000 nm (Goldcamp et al., c2003).
Cu	Cu is entered roots as monovalent ions, transported to the leaves in the form of Cu(I) or Cu(II) complexes, and reduced to Cu^+ before entering the leaf cell (Printz et al., 2016). In a Co hyperaccumulator, *Haumaniastrum katangense*, the chemical speciation of Cu is associated with carboxylic acids van der Ent et al. (2019). Cu^+ has a d^{10} configuration, and thus no absorption bands are expected.
Zn	Zn enters roots either as Zn^{2+} or a Zn organic complex (Broadley et al., 2007). In many crop species, Zn is found to form insoluble complexes with phosphate (P) such as $Zn_3(PO4)_2$ that is Zn^{2+} and Zn-phytates (Sarret et al., 2002; Zhao et al., 1998, 2000). In Zn hyperaccumulator plant species, *Thlaspi caerulescens*, Zn is believed to for a complex with malate acids (Tolrà et al., 1996). Zn^{2+} has a d^{10} configuration, and thus no absorption bands are expected.
Nd	The ionic radius (85–115 pm) of the REEs trivalent cation is similar to the Ca divalent cation (99 pm). The trivalent cations of the REEs are analogs to Ca^{2+}, and found to be located at the Ca binding sites in organisms, thus showing a similar effect with that of Ca (Wang et al. 1998). The uptake of the lanthanide trivalent cation is regarded to follow the pathway of Ca^{2+}(Liu et al. 2012). Neodymium $(Nd)^{3+}$ absorption bands (at ~744 nm, ~802 nm, and ~871 nm) dominate the reflectance spectra of REEs-bearing minerals such as monazite and britholite (Turner et al. 2016), and the Nd^{3+} absorption feature in REEs bearing minerals can be observed when Nd concentration is more than 1000 mg Kg^{-1}(Neave et al. 2016).

References

Akabayov SR, Akabayov B (2014) Vanadate in structural biology. Inorganica Chim Acta 420:16–23. https://doi.org/10.1016/j.ica.2014.02.010

Blamey FPC, McKenna BA, Li C, et al (2018) Manganese distribution and speciation help to explain the effects of silicate and phosphate on manganese toxicity in four crop species. New Phytol 217:1146–1160. https://doi.org/10.1111/nph.14878

Broadley MR, White PJ, Hammond JP, et al (2007) Zinc in plants. New Phytol 173:677–702. https://doi.org/10.1111/j.1469-8137.2007.01996.x

Collins RN, Bakkaus E, Carrière M, et al (2010) Uptake, localization, and speciation of cobalt in *triticum aestivum l.* (Wheat) and lycopersicon esculentum M. (Tomato). Environ Sci Technol 44:2904–2910. https://doi.org/10.1021/es903485h

Crans DC, Smee JJ, Gaidamauskas E, Yang L (2004) The chemistry and biochemistry of vanadium and the biological activities exerted by vanadium compounds. Chem Rev 104:849–902. https://doi.org/10.1021/cr020607t

Fernando DR, Mizuno T, Woodrow IE, et al (2010) Characterization of foliar manganese (Mn) in Mn (hyper)accumulators using x-ray absorption spectroscopy. New Phytol 188:1014–1027. https://doi.org/10.1111/j.1469-8137.2010.03431.x

Goldcamp MJ, Edison SE, Squires LN, et al (2003) Structural and spectroscopic studies of nickel(II) complexes with a library of bis(oxime)amine-containing ligands. Inorg Chem 42:717–728. https://doi.org/10.1021/ic025860q

Huffman EWD, Allaway WH (1973) Chromium in plants: distribution in tissues, organelles, and extracts and availability of bean leaf cr to animals. J Agric Food Chem 21:982–986. https://doi.org/10.1021/jf60190a008

Jones MR, Luther GW, Mucci A, Tebo BM (2019) Concentrations of reactive Mn(III)-L and MnO2 in estuarine and marine waters determined using spectrophotometry and the leuco base, leucoberbelin blue. Talanta 200:91–99. https://doi.org/10.1016/j.talanta.2019.03.026

Kasai M, Yamazaki J, Kikuchi M, et al (1999) Concentration of vanadium in soil water and its effect on growth and metabolism of rye and wheat plants. Commun Soil Sci Plant Anal 30:971–982. https://doi.org/10.1080/00103629909370261

Lee J, Reeves RD, Brooks RR, Jaffré T (1977) Isolation and identification of a citrato-complex of nickel from nickel-accumulating plants. Phytochemistry 16:1503–1505. https://doi.org/10.1016/0031-9422(77)84010-7

Liu D, Wang X, Chen X, et al (2012) Effects of lanthanum on the change of calcium level in the root cells of rice. Commun Soil Sci Plant Anal 43:1994–2003. https://doi.org/10.1080/00103624.2012.693231

Lopez-Millan AF, Morales F, Abadia A, Abadia J (2000) Effects of iron deficiency on the composition of the leaf apoplastic fluid and xylem sap in sugar beet. Implications for iron and carbon transport. Plant Physiol 124:873–884. https://doi.org/10.1104/pp.124.2.873

Matzapetakis M, Dakanali M, Raptopoulou CP, et al (2000) Synthesis, spectroscopic, and structural characterization of the first aqueous cobalt(II)-citrate complex: Toward a potentially bioavailable form of cobalt in biologically relevant fluids. J Biol Inorg Chem 5:469–474. https://doi.org/10.1007/s007750050007

Mishra D, Kar M (1974) Nickel in plant growth and metabolism. Bot Rev 40:395–452. https://doi.org/10.1007/BF02860020

Neave DA, Black M, Riley TR, et al (2016) On the feasibility of imaging carbonatite-hosted rare earth element deposits using remote sensing. Econ Geol 111:641–665. https://doi.org/10.2113/econgeo.111.3.641

Oven M, Grill E, Golan-Goldhirsh A, et al (2002) Increase of free cysteine and citric acid in plant cells exposed to cobalt ions. Phytochemistry 60:467–474. https://doi.org/10.1016/S0031-9422(02)00135-8

Palit S, Sharma A, Talukder G (1994) Effects of cobalt on plants. Bot Rev 60:149–181. https://doi.org/10.1007/BF02856575

Printz B, Lutts S, Hausman JF, Sergeant K (2016) Copper trafficking in plants and its implication on cell wall dynamics. Front Plant Sci 7:1–16. https://doi.org/10.3389/fpls.2016.00601

Shanker AK, Cervantes C, Loza-Tavera H, Avudainayagam S (2005) Chromium toxicity in plants. Environ Int 31:739–753. https://doi.org/10.1016/j.envint.2005.02.003

Tolrà RP, Poschenrieder C, Barceló J (1996) Zinc hyperaccumulation in *Thlaspi caerulescens.* II. Influence on organic acids. J Plant Nutr 19:1541–1550. https://doi.org/10.1080/01904169609365220

Turner DJ, Rivard B, Groat LA (2016) Visible and short-wave infrared reflectance spectroscopy of REE phosphate minerals. Am Mineral 101:2264–2278. https://doi.org/10.2138/am-2016-5692

Van Der Ent A, Malaisse F, Erskine PD, et al (2019) Abnormal concentrations of Cu-Co in: *Haumaniastrum katangense, Haumaniastrum robertii and Aeolanthus biformifolius*: Contamination or hyperaccumulation? Metallomics 11:586–596. https://doi.org/10.1039/c8mt00300a

Wang W, C T, Wang H (1998) Research advance on interaction between metal ion of REEs and enzyme molecule. Chinese Rare Earths (in Chinese) 19:57–65
